# Point-of-care ultrasound in primary care – EFSUMB core curriculum and training recommendations – a position paper

**DOI:** 10.1055/a-2771-2848

**Published:** 2026-04-28

**Authors:** Camilla Aakjær Andersen, Christian Jenssen, Aaron Poppleton, Eva Leceaga, Mateusz Kosiak, Mihai Sorin Iacob, Mariela Skendi, Thomas Frese, Thomas Løkkegaard, Vesna Homar, Viktor Rüttermann, Caroline Ewertsen

**Affiliations:** 1 Center for General Practice at Aalborg UniversityAalborg University Faculty of MedicineGistrupDenmark; 2 EFSUMB – European Federation of Societies for Ultrasound in Medicine and BiologyLondonUnited Kingdom of Great Britain and Northern Ireland; 3 Department of Internal Medicine 116976 Krankenhaus Märkisch-Oderland GmbHStrausberg/WriezenGermany; 4 Medical University BrandenburgBrandenburg Institute of Clinical Ultrasound (BICUS)NeuruppinGermany; 5 School of Medicine 4212 Keele UniversityNewcastle-under-LymeEnglandUnited Kingdom of Great Britain and Northern Ireland; 6 EFMUS – The European Family Medicine Ultrasound SocietyUnited Kingdom of Great Britain and Northern Ireland; 7 Primary Care Center Ronda Prim, Direction of Primary Health Care Barcelones North and Maresme 37494 Catalan Institute of HealthMataróSpain; 8 EcoAP Working Group on Ultrasound in Primary Care 636307 Catalan Society of Family and Community Medicine AssocBarcelonaSpain; 9 EYFDM – European Young Family Doctors MovementSlovenia; 10 Puck Medical CentrePuckPoland; 11 eduson.pl – Sonographic educational platform for physicians and medical studentsKrosinkoPoland; 12 Centre for Preventive Medicine 162271 University of Medicine and Pharmacy Victor Babes TimisoaraTimisoaraRomania; 13 EUVEKUS – European Ultrasound Working Group in Ambulatory HealthcareViennaAustria; 14 Department of General Practice 27063 Sorbonne UniversityParisÎle-de-FranceFrance; 15 SNECHO-MG – French Society for Ultrasound in General MedicineFrance; 16 Francophone Society of UltrasoundFrance; 17 Institute of General Practice and Family Medicine 9176 Martin-Luther-Universitat Halle-WittenbergHalleSachsen-AnhaltGermany; 18 Wonca Europe 416116 World Organization of National Colleges, Academies and Academic Associations of General Practitioners/Family PhysiciansSlovenia; 19 DAUS – Danish Society for Ultrasound in General PracticeDenmark; 20 Department of Family MedicineFaculty of MedicineUniversity of LjubljanaLjubljanaSlovenia; 21 30895 EURACT – European Academy of Teachers in General Practice/Family MedicineSwitzerland; 22 Hausärztliche Gemeinschaftspraxis Nordholter WegMedical Practice DrensteinfurtDrensteinfurtGermany; 23 DEGUM – German Society for Ultrasound in Medicine and Biology, Working Group Primary CareGermany; 24 Department of RadiologyCopenhagen University Hospital, RigshospitaletCopenhagen OEDenmark

**Keywords:** Education, Training, Quality Assurance, Point of care, Primary care, General Practice

## Abstract

Frontline physicians working in primary care increasingly use diagnostic ultrasound examinations and simple ultrasound-guided procedures as part of their daily practice. Primary care is organized in many ways across Europe and as a result, primary care physicians have different qualifications in terms of using and integrating point-of-care ultrasound in patient care. To ensure high quality and standardized practice across Europe, this position paper of the European Federation of Societies for Ultrasound in Medicine and Biology (EFSUMB) describes training recommendations and a generic core curriculum for frontline physicians working in primary care. The core curriculum was developed through a Delphi process and includes basic ultrasound examinations corresponding to the EFSUMB competence level 1 for medical ultrasound practice. The training recommendations are intended to build a common foundation while national adjustments must be made to ensure relevance in the clinical setting and patient population.

## Zusammenfassung

In der Primärversorgung tätige Ärzte setzen zunehmend diagnostische Ultraschalluntersuchungen und einfache ultraschallgesteuerte Verfahren als Teil ihrer täglichen Routine ein. Die Primärversorgung ist in Europa sehr unterschiedlich organisiert, sodass die Qualifikationen der Ärzte, die Point-of-Care-Ultraschall in der primären Patientenversorgung einsetzen und integrieren, sehr unterschiedlich sind. Mit dem Ziel, eine hohe Qualität und standardisierte Ausübungspraxis in ganz Europa zu gewährleisten, stellt dieses Positionspapier der European Federation of Societies for Ultrasound in Medicine and Biology (EFSUMB) Ausbildungsempfehlungen und ein PoCUS Basis-Curriculum für in der Primärversorgung tätige Ärzte vor. Das Basis-Curriculum wurde im Rahmen eines Delphi-Verfahrens entwickelt und umfasst grundlegende Ultraschalluntersuchungen, die dem EFSUMB-Kompetenzniveau 1 für die medizinische Ultraschallpraxis entsprechen. Die Ausbildungsempfehlungen sollen eine gemeinsame Grundlage schaffen, wobei durch nationale Anpassungen die Relevanz für das konkrete klinische Umfeld und die betreuten Patientengruppen sichergestellt werden muss.

## Introduction

Point-of-care ultrasound (PoCUS) is defined as an ultrasound examination performed at the bedside by the treating physician to answer a specific clinical question in real-time as part of a clinical examination 1 2 3 . These examinations are usually focused and simple examinations performed in a targeted manner to rule in or rule out specific conditions. This makes them less time-consuming than traditional comprehensive examinations exploring an entire anatomical region 4 5 . PoCUS is increasingly used by frontline physicians working in primary care 6 7 as a result of the increased availability of mobile ultrasound systems and the rapidly growing evidence supporting the integration of selected PoCUS examinations as a first-line imaging modality across medical specialties 8 9 10 11 12 13 14 .

The way PoCUS is integrated and used reflects differences in the organization of primary healthcare systems across countries 15 . In some countries, primary care physicians work as generalists seeing a broad unselected patient population, while in other countries primary care physicians subspecialize and subsequently see selected patient groups 16 . The training background of primary care physicians also varies across countries 17 and not all physicians are trained in ultrasound during their undergraduate training or specialization 18 . This means that ultrasound is currently used in different ways in primary care – both for simple PoCUS and for more advanced ultrasound examinations – and by frontline physicians with different ultrasound skillsets.

Nonetheless, ultrasound is a user-dependent technology, and operators must have the ability to systematically integrate anatomical and clinical knowledge with visual-spatial coordination, technological understanding, and image recognition. This highlights the importance of sufficient training to ensure competence and correct interpretation 19 . In addition, some ultrasound examinations are more difficult to perform than others, have a low diagnostic accuracy in the hands of generalists, or entail a higher risk of misinterpretation, under- and overdiagnosis, incidental findings, and ultimately harm to patients 20 21 22 . Therefore, choosing appropriate applications is paramount 23 . However, the appropriateness of applications will depend on the specialization of the examiner and the context in which PoCUS is used. Therefore, the appropriateness will differ between PoCUS performed by GPs and clinical specialists and PoCUS performed in emergency situations 24 25 , at hospitals 26 , in a clinic-based general practice setting 27 , and during home visits for geriatric and palliative care patients 28 .

In some countries, ultrasound is already integrated into the training and clinical practice of primary healthcare doctors 29 , while in other countries there are significant barriers to implementation 30 31 32 . The European Federation of Societies for Ultrasound in Medicine and Biology (EFSUMB) aims to make medical ultrasound a reliable professional service based on common and uniform quality standards across medical specialties and healthcare systems 33 . Therefore, the EFSUMB defined the creation of a uniform core curriculum for frontline physicians working in primary care as one of its strategic goals 34 with the purpose of supporting efforts to establish PoCUS training in European countries and to develop the basis for a common standard in this important part of clinical ultrasound diagnostics.

## Background

This EFSUMB position paper describes the training requirements for the primary care PoCUS core curriculum. The training recommendations are based on EFSUMB’s fundamental statements 33 and follow the general principles of three competency levels for the practice of medical ultrasound used by the EFSUMB and the Royal College of Radiologists in the United Kingdom 35 36 , which represent a gradual increase in skills, experience, and competence.

The EFSUMB core curriculum has been developed through a Delphi process involving 95 primary care doctors with more than two years of ultrasound experience and experience teaching ultrasound to others. The primary care doctors originated from 28 European countries representing different healthcare organizations and medical specialties 37 . A list of 232 different diagnostic ultrasound examinations and eight ultrasound-guided procedures formed the foundation for the Delphi process. Through consecutive rounds, consensus was reached with respect to including 40 diagnostic ultrasound examinations and no ultrasound-guided procedures in a core curriculum for frontline physicians working in primary care.

According to the applied selection criteria in the Delphi process, the 40 diagnostic ultrasound examinations correspond to EFSUMB level 1 competency 35 and the training recommendations have been developed accordingly.

The training recommendations were developed based on the best-available evidence on the learning process for primary care physicians 38 39 40 and experiences within an appointed EFSUMB task force including experts in primary care and imaging (listed authors).

The training recommendations have an emphasis on skill development over time and scaffolded continuous practice including feedback on performance in line with the findings in a previous systematic review 38 . Fig. 1 depicts the basic learning trajectory.

**Fig. 1:**
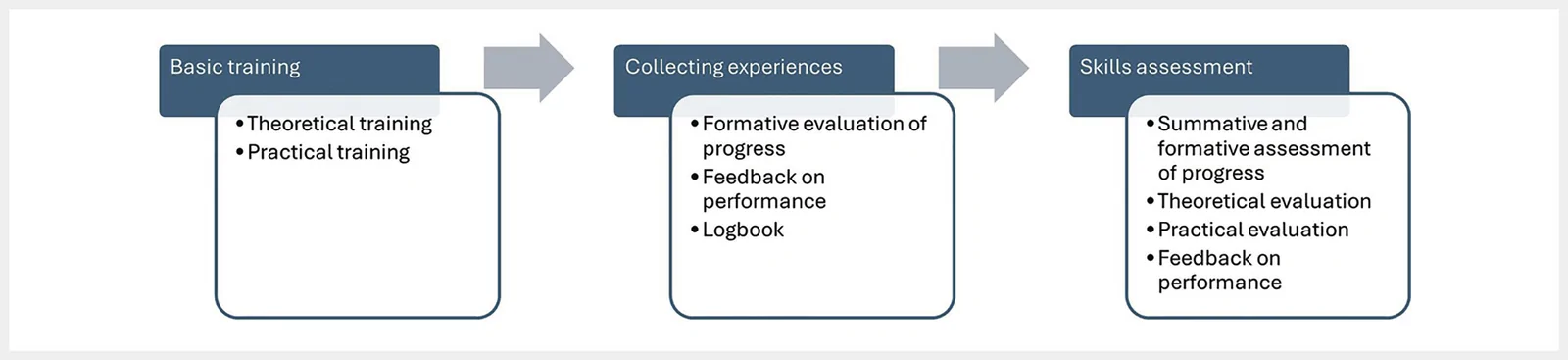
Skills development trajectory for obtaining basic scanning competence.

As learning curves differ between individuals 41 and evidence for the application of a specific cut-off is sparse, the recommendations include no predefined minimum number of examinations or number of training hours as an indicator of competency. Instead, competency-based education using both formative and summative means of evaluation to determine competence, create self-awareness, and ensure sufficient quality is recommended.

In line with the EFSUMB minimum training recommendations for medical practice of ultrasound 35 , we recommend that ultrasound training for frontline physicians working in primary care includes both theoretical knowledge and practical skills. We recommend that ultrasound training is not restricted to the technical ability to produce ultrasound images but also includes training regarding indications for performing the examination, interpretation of ultrasound findings, recognition of scanning limitations, and integration of findings in the clinical context 42 . Frontline physicians using ultrasound must acknowledge their individual limitations based on their own level of experience, their clinical background, and the ultrasound technology being used and decide when to request support from more experienced colleagues or secondary care specialists – or to consider other imaging modalities.

We have outlined the recommended basic theoretical knowledge and skills with a description of the corresponding training and assessment requirements for clinical practices. Each corresponds with the recommended core curriculum, with the scope of practice being defined for each application.

## Training recommendations

To meet the EFSUMB level 1 standard 35 , the trainee should demonstrate basic knowledge of the normal and pathological ultrasound anatomy of the relevant structures corresponding to the EFSUMB core curriculum and practical skills with regard to performing a systematic PoCUS examination, image interpretation, documentation, optimal clinical integration, and medical decision making based on the ultrasound examination 42 . The general knowledge and competences to be acquired are outlined in Fig. 2 , while application-specific knowledge and competences are listed in Table 1 .

**Fig. 2:**
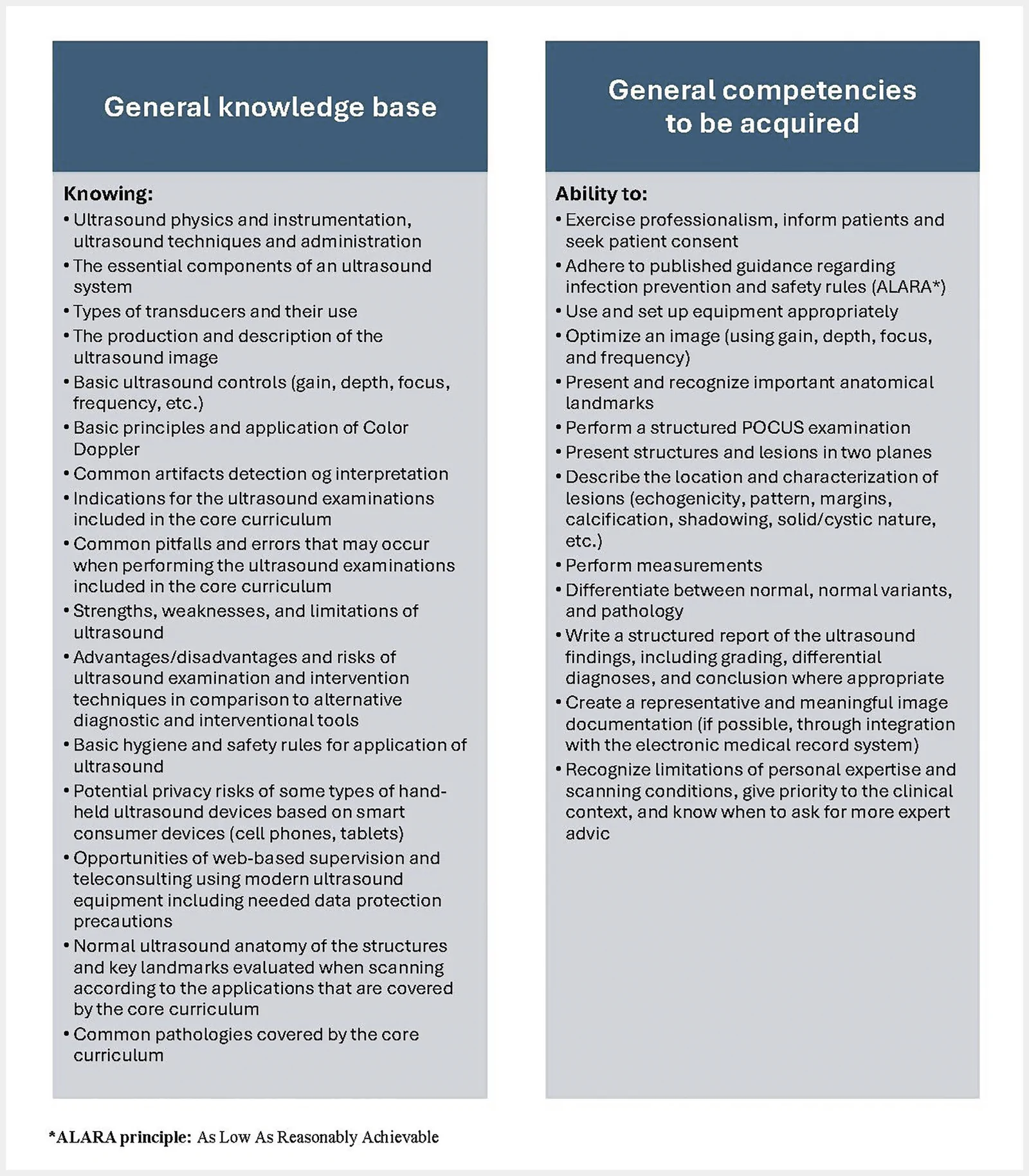
General Knowledge and competences to be obtained.

**Table:** **Table 1** Application-specific knowledge and competences to be acquired.

Condition to be ruled in/out using POCUS	Application-specific knowledge	Application-specific competences
**Vascular**		
**Aorta** (abdominal aortic aneurysm)	Know the normal anatomy and ultrasound appearance of the abdominal aorta Know normal diameters and risk related to diameters and consequences (follow-up intervals, involving a vascular surgeon) Know the location of specific ultrasound landmarks Know the ultrasound characteristics of an abdominal aneurysm	Ability to obtain correct imaging and scan through the aorta in the transverse and longitudinal planes Ability to obtain correct measurements Ability to determine the presence or absence of an aneurysm and determine its extension
**Lower limb veins** (deep vein thrombosis)	Know the normal anatomy and location of the deep leg veins including their branches and relationship to the artery Know the location of specific ultrasound landmarks Know the ultrasound characteristics of deep vein thrombosis	Ability to obtain correct imaging and scan through the common femoral vein, femoral vein, and popliteal vein until it bifurcates Ability to use color Doppler to differentiate between vessels and visualize flow direction Ability to detect signs of a deep vein thrombosis (visualizable thrombus in the vein, un-compressible vein)
**Abdomen**		
**Abdomen** (intraperitoneal fluid)	Know the normal anatomy of the abdomen and the places for intraperitoneal fluid accumulation. Know the location of specific ultrasound landmarks for the detection of intraperitoneal fluid accumulation.	Ability to obtain correct imaging for the assessment of fluid accumulation in the RUQ (hepatorenal space/Morison’s pouch, caudal tip of the liver, supra-hepatic space), LUQ (peri-splenic space, spleen tip, splenorenal recess), and pelvis (the rectovesical pouch (male), the rectouterine pouch Douglas (female)). Ability to detect signs of ascites/intraperitoneal bleeding.
**Gallbladder** (gallbladder stone, cholecystitis, congested gallbladder, gallbladder wall polyp, dilatation of intrahepatic bile ducts)	Know the normal anatomy and ultrasound appearance of the gallbladder and extra- and intrahepatic bile ducts Know typical anatomical variants of the gallbladder including appearance in fasting state or after meal/smoking Know the location of specific ultrasound landmarks in relation to the gallbladder and bile ducts Know the ultrasound characteristics of the liver parenchyma, the portal vein, the common bile duct, and the gall bladder Know cut-off values for gallbladder and extrahepatic bile duct diameter Know typical ultrasound criteria for acute cholecystitis	Ability to obtain correct imaging and scan through the gallbladder in the transverse and longitudinal plane Ability to obtain the correct measurement of the anterior gallbladder wall and of the longitudinal and transverse diameter of the gallbladder Ability to detect and differentiate between a gallbladder stone and a gallbladder wall polyp Ability to detect signs of biliary dilatation
Liver (steatotic liver, liver cyst, hepatomegaly)	Know the normal anatomy and ultrasound appearance of the liver Know the location of specific ultrasound landmarks in relation to the liver Know the ultrasound characteristics of the liver parenchyma, the portal vein, the common bile duct, and the gall bladder Know the typical ultrasound criteria of the steatotic liver Know the basic criteria defining a focal liver lesion, in particular a liver cyst	Ability to obtain correct imaging and scan through the liver in the transverse and longitudinal plane Ability to determine the presence or absence of hepatomegaly Ability to assess the echogenicity of the liver parenchyma in comparison with the kidney and other useful criteria (attenuation, vascular blurring) to determine the presence or absence of steatosis Ability to detect and describe simple cystic lesions and to differentiate them from complex cystic lesions and from solid lesions
**Spleen** (enlarged spleen)	Know the normal anatomy and ultrasound appearance of the spleen Know the location of specific ultrasound landmarks to enable correct measurements Know the ultrasound characteristics of the normal splenic parenchyma	Ability to acquire correct imaging and scan through the spleen in the transverse and longitudinal plane Ability to obtain the correct measurement to determine the size of the spleen Ability to determine the presence or absence of an enlarged spleen
**Urinary tract**		
**Kidney** (hydronephrosis, kidney stones, focal simple cystic lesion, ureteral obstruction)	Know the ultrasound anatomy of the kidney and ureter in different age groups Know where to look for a dilated ureter Know the ultrasound characteristics of common kidney pathologies (hydronephrosis, kidney stones, focal lesions including cysts) Know the location of common kidney pathologies	Ability to obtain full visualization of both kidneys including the ability to sweep through the organ in both the longitudinal and the transverse plane Ability to determine the presence or absence of hydronephrosis and dilated ureter including grading the severity of obstruction Ability to detect and describe the presence of simple cystic lesions and to differentiate them from complex cystic lesions and from solid lesions
**Urinary bladder** (residual urine, Foley catheter, Stones)	Know the ultrasound anatomy of the urinary bladder and ureters Know the ultrasound characteristics of adjacent structures and landmarks including the prostate and uterus Know the location of common urinary bladder pathologies and the ultrasound characteristics of a catheter	Ability to acquire images of the urinary bladder in the transverse and longitudinal plane Ability to make correct measurements and determine bladder volume Ability to detect the location of a Foley catheter Ability to detect and describe the presence of common abnormalities in the bladder (prostate hypertrophy, bladder stones)
**Thorax**		
**Heart** (pericardial effusion/cardiac tamponade)	Know the ultrasound anatomy of the heart in the subxiphoid view Know the location of the descending aorta Know the location of pericardial effusion Know the sign of cardiac tamponade	Ability to acquire the subxiphoid image of the heart, visualizing all four chambers Ability to determine the presence or absence of fluid accumulation in the pericardial sac Ability to detect signs of cardiac tamponade
**Pleura and lungs** (pleural effusion or hemothorax, pneumothorax, interstitial syndrome, pneumonia)	Know the ultrasound anatomy of the lungs and pleura Know the normal ultrasound findings (lung sliding, A-lines, lung pulse, curtain sign, seashore sign) and where to look for specific artifacts and landmarks Know the ultrasound signs of abnormalities and pathologies in relation to parietal pleura and pleural cavities (pleural thickening, calcifications, effusion and pneumothorax: lung point, barcode sign) Know the ultrasound signs of abnormalities and pathologies in relation to visceral pleura and lungs (B-lines as focal pattern or interstitial syndrome, pleural thickening, pleural fragmentation, comet tail artifacts, consolidation, air bronchogram, fluid bronchogram)	Ability to choose the optimal patient positioning and ultrasound mode (B-mode, M-mode) for a given clinical context Ability to obtain images of the pleura and the lungs preferably from the anterior, posterior, left and right sides using a systematic approach Ability to detect/exclude fluid in the pleural cavity and describe the location and characterization of pleural effusions (semiquantitative size estimation, echogenicity, septations) Ability to detect signs of pneumothorax (absence of lung sliding/absence of lung pulse/lung point/ barcode sign) Ability to describe the location and characterization of the visceral pleural and lung abnormalities (focal B-lines/ comet tail artifacts, interstitial syndrome, thickened and/or fragmented pleura, consolidations, air and fluid bronchogram)
**MSK and soft tissue**		
**Knee** (joint effusion, Baker’s cyst)	Know the ultrasound anatomy of the knee and the location of the synovial recesses of the knee Know the ultrasound characteristics of adjacent structures and landmarks Know the location of knee effusion and Baker’s cyst	Ability to obtain images of the suprapatellar recess anterior and posterior to the space between the semimembranosus and medial gastrocnemius tendons Ability to detect effusions and access the amount of fluid accumulation
**Achilles tendon** (rupture)	Know the ultrasound anatomy of the Achilles tendon Know the ultrasound signs of tendinopathy (tendon thickness, echogenicity and vascularity), partial and complete tendon ruptures	Ability to obtain images of the Achilles tendon including the calcaneal insertion and scan through the tendon in the longitudinal and transverse plane Ability to detect and describe a contour change of the tendon, partial or complete discontinuation of the tendon’s fibrillar echotexture, or a gap between fibers
**Soft tissue** (subcutaneous abscess, foreign body, hematoma, phlegmon/cellulitis, fibrolipoma)	Know the ultrasound anatomy of the skin and soft tissue Know the ultrasound signs of bleeding, infection, and inflammation in soft tissue Know the ultrasound appearance of different foreign bodies that have penetrated the skin Know the ultrasound characteristics of a fibrolipoma and know when to refer a patient with a soft-tissue lump for further imaging or follow-up	Ability to obtain images of superficial soft tissue in two planes Ability to detect, assess, and describe foreign bodies or soft-tissue collections, echogenicity, and vascularity Ability to make the correct assessment and measurements and to describe size, margins, vascularity, location, and echo pattern of fibrolipomas
**Neck** (pathological lymph nodes)	Know the ultrasound anatomy and location of cervical lymph nodes Know the basic ultrasound characteristics of enlarged or pathological lymph nodes	Ability to acquire images of superficial soft-tissue masses in two planes Ability to make correct measurements and detect enlarged lymph nodes Ability to describe and characterize the enlarged lymph nodes in terms of size, site, shape, borders, echogenicity, vascularity, any adjacent soft-tissue edema, and internal architecture of the lymph nodes (loss of hilar architecture, cortical thickening, presence of intranodal necrosis, and calcification)
**Pelvis**		
**Female pelvis** (intrauterine pregnancy, fetal heartbeat, fluid within the cul-de-sac, Position of intrauterine contraceptive device)	Know the ultrasound anatomy of the uterus and cul-de-sac Know the variations in ultrasound appearance throughout the menstrual cycle and pregnancy Know the ultrasound signs of early pregnancy (gestational sac, yolk sac, embryo, fetal heartbeat) and their appearance according to gestational age Know the correct position of an intrauterine contraceptive device (IUCD) in the uterus and the ultrasound characteristics of different devices	Ability to obtain transabdominal and transvaginal images of the uterus and scan through the uterus in the longitudinal and transverse plane Ability to detect and characterize the location and appearance of the uterus Ability to detect fluid accumulation in the cul-de-sac beyond expected physiological variation Ability to detect and characterize signs of early pregnancy and relate ultrasound findings to the expected gestational age Ability to detect and characterize the location of an IUCD
**Male pelvis** (prostate hypertrophy)	Know the ultrasound anatomy of the prostate Know the ultrasound signs and volume indicating prostate hypertrophy	Ability to obtain images of the prostate transabdominally and scan through the organ in the longitudinal and transverse plane Ability to accurately measure the volume of the prostate

A basic ultrasound course for frontline physicians should be divided into structured modules based on anatomy or functional applications, rather than presenting all 40 scans from the Core Curriculum at once. This modular approach facilitates training, supports the gradual development of skills, and allows for customization to align with national or regional healthcare needs.

For EFSUMB level 1, we recommend a teaching approach in which theoretical and practical training is delivered over time and is fully integrated with ongoing formative evaluation and feedback on performance in each training step as described in Fig. 3 . A comprehensive evidence-based basic training program including all of these steps has been developed and described 39 , but national adaptations can be made to incorporate the ultrasound training into residency, specialty training, fellowship training, or continuing medical education (CME) courses. Regardless, we recommend a longitudinal and stepwise approach starting with the simple applications in order to build a foundation of knowledge before increasing complexity. E-learning tools can be incorporated into the teaching of ultrasound fundamentals and as interactive elements of the curriculum, but such tools cannot replace hands-on training. We recommend that at least 50% of the training must be spent with practical elements, especially hands-on training, live demonstrations, and interactive case discussions. For hands-on training, a maximum of five trainees should share one ultrasound system or simulator and should be supervised by one tutor 43 44 . Fig. 3 outlines our recommendations in nine steps building on the best-available evidence and fundamental teaching principles 35 36 37 38 39 45 46 47 48 .

**Fig. 3:**
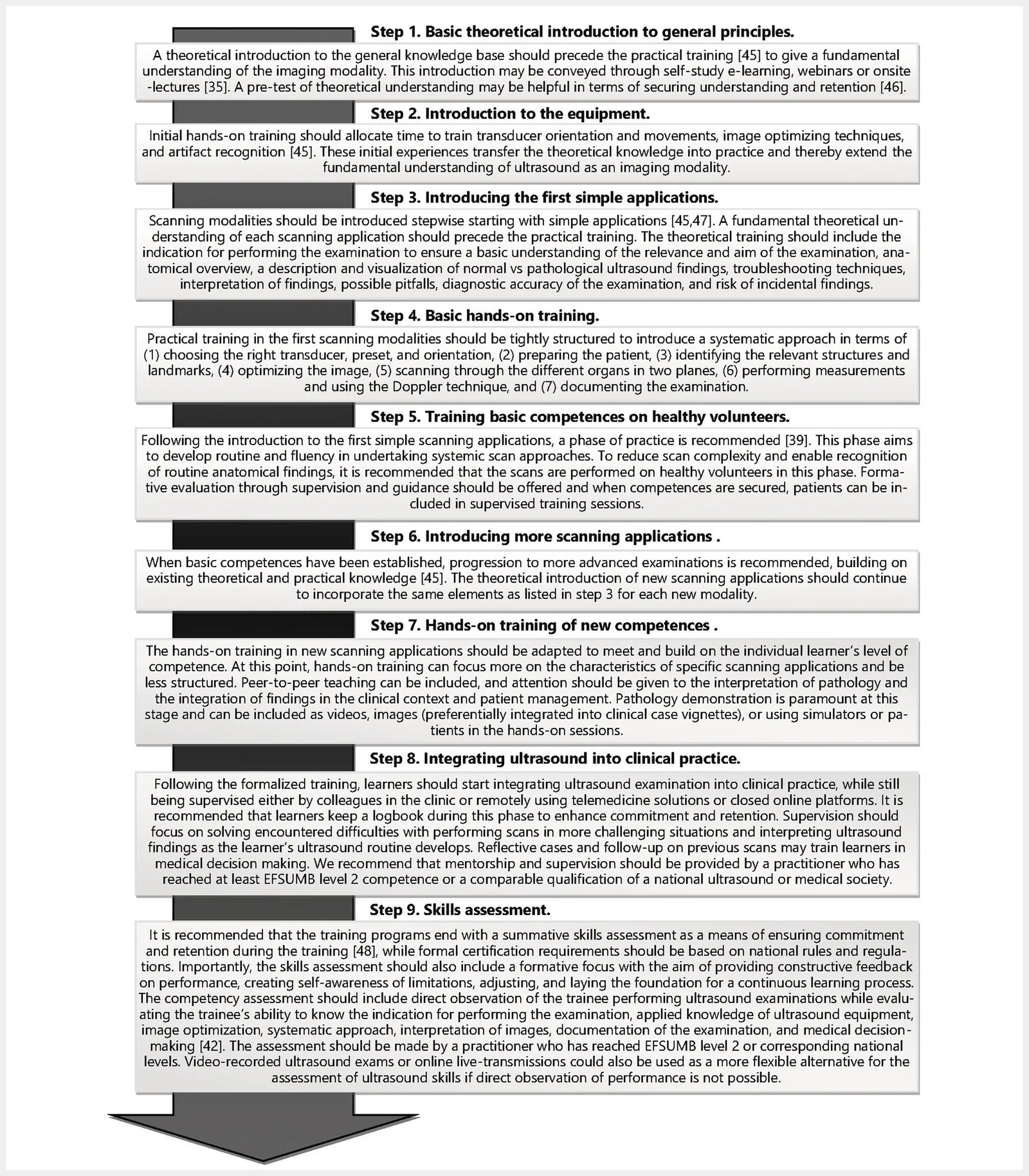
Nine steps in a basic ultrasound training program.

We recommend that course providers, organizing basic ultrasound training for primary care/frontline physicians, structure the course in the nine steps and bear the responsibility for the final skills assessment to ensure that learners reach the desired outcomes and complete the training.

## Adjusting the core curriculum to national settings and specific needs

We recommend that a basic ultrasound course aimed at frontline physicians predominantly include scans outlined in the Basic Core Curriculum. However, as primary care is organized differently across Europe, frontline physicians working in primary care can have different medical qualifications and work conditions and encounter different patient populations. In the Delphi process for the core curriculum development, some simple applications specifically suited for, e.g., pediatricians were deselected owing to the small number of pediatricians (N=4) participating in the panel 37 . Hence, the core curriculum and the corresponding training recommendations must be adjusted to the national setting to meet local needs. In addition, modular extensions according to specialties and special interests (e.g., primary care pediatricians, palliative care physicians performing home visits, out-of-hours emergency care services) are possible. However, the core curriculum has been designed to include scanning modalities for generalists requiring a basic level of scanning competence and the training recommendations have been developed based on the best-available evidence for ultrasound training of novices. Therefore, they are thought to be largely applicable across healthcare systems. In the future, artificial intelligence may offer new and less resource-demanding solutions for guidance, feedback, quality assurance and skills assessment, but until the technology is validated and widely available, we recommend that basic ultrasound training of frontline physicians – across healthcare systems – include longitudinal training with proctored scans and support by more experienced clinical colleagues or sonographers.

## Maintenance of ultrasound skills

Practitioners should regularly, i.e., on a weekly basis, perform or supervise ultrasound examinations to further consolidate skills and maintain competence. This should be in compliance with local regulations, continuing professional development, and the updating of practice. Besides ensuring that regulations are met, documentation and image storage may serve as a learning source for quality assurance and further development. An audit of ultrasound practices can even be included in countries performing a routine annual appraisal. Attendance at appropriate ultrasound courses or conferences together with regular reviews of the current literature is expected. It is recommended that operators who experience prolonged periods of time without exposure to ultrasound attend courses or seek supervision prior to re-starting the clinical use of ultrasound. In some countries, national ultrasound societies offer extended course content for GPs and other primary care physicians to provide knowledge and skills in more comprehensive ultrasound applications, using PoCUS applications as a foundation. Frontline physicians, who have completed training on a curricular basis and practice ultrasound regularly are strongly encouraged to take advantage of these structured training opportunities.

## Conclusion

The use of ultrasound by frontline physicians in primary care is becoming increasingly prevalent. These training recommendations use current evidence and clinical experience to guide physicians and healthcare systems with regard to the implementation of basic ultrasound examinations. The training recommendations advise structuring ultrasound education in nine steps in which competencies are built over time and including elements of both formative and summative evaluation.

## References

[1] Dietrich CF Goudie A Chiorean L Point of Care Ultrasound: A WFUMB Position Paper Ultraschall in Med 2017 43 01 49 58 10.1016/j.ultrasmedbio.2016.06.021 27472989

[2] Patel MD Mapping the Ultrasound Landscape to Define Point-of-Care Ultrasound and Diagnostic Ultrasound: A Proposal From the Society of Radiologists in Ultrasound and ACR Commission on Ultrasound J Am Coll Radiol 2021 18 01 42 52 10.1016/j.jacr.2020.09.013 33007309

[3] Oto B Baeten R Chen L Best Practices for Point of Care Ultrasound: An Interdisciplinary Expert Consensus POCUS J 2024 9 01 95 108 10.24908/pocus.v9i1.17240 38681157 PMC11044939

[4] Díaz-Gómez JL Mayo PH Koenig SJ Point-of-care ultrasonography NEJM 2021 385 1593 1602 10.1056/NEJMra1916062 34670045

[5] Weile J Brix J Moellekaer AB Is point-of-care ultrasound disruptive innovation? Formulating why POCUS is different from conventional comprehensive ultrasound Crit Ultrasound J 2018 10 01 25 10.1186/s13089-018-0106-3 PMC6165827 30270416

[6] Myklestul HC Skonnord T Brekke M Point-of-care ultrasound (POCUS) in Norwegian general practice Scand J Prim Health Care 2020 38 02 219 225 10.1080/02813432.2020.1753385 32314640 PMC8570758

[7] Touhami D Merlo C Hohmann J The use of ultrasound in primary care: longitudinal billing and cross-sectional survey study in Switzerland BMC Family Practice 2020 21 1 11 10.1186/s12875-020-01209-7 PMC7330951 32611390

[8] Szabó GV Szigetváry CSzabó L Point-of-care ultrasound improves clinical outcomes in patients with acute onset dyspnea: a systematic review and meta-analysis Intern Emerg Med 2023 18 02 639 653 10.1007/s11739-022-03126-2 PMC10017566 36310302

[9] Sorensen B Hunskaar S Point-of-care ultrasound in primary care: a systematic review of generalist performed point-of-care ultrasound in unselected populations Ultrasound J 2019 11 01 31 10.1186/s13089-019-0145-4 31749019 PMC6868077

[10] Bøtker MT Jacobsen L Rudolph SS The role of point of care ultrasound in prehospital critical care: a systematic review Scand J Trauma Resusc Emerg Med 2018 26 01 51 10.1186/s13049-018-0518-x. 29940990 PMC6019293

[11] Stone BS Muruganandan KM Tonelli MM Impact of point-of-care ultrasound on treatment time for ectopic pregnancy Am J Emerg Med 2021 49 226 232 10.1016/j.ajem.2021.05.071 34146921

[12] Poppleton A Tsukagoshi S Vinker S World Organization of National Colleges, Academies and Academic Associations of General Practitioners and Family Physicians (WONCA) Europe position paper on the use of point-of-care ultrasound (POCUS) in primary care Prim Health Care Res Dev 2024 25 e21 10.1017/S1463423624000112 38651341 PMC11091537

[13] Zaki HA Albaroudi B Shaban EE Deep venous thrombosis (DVT) diagnostics: gleaning insights from point-of-care ultrasound (PoCUS) techniques in emergencies: a systematic review and meta-analysis Ultrasound J 2024 16 01 37 10.1186/s13089-024-00378-1 PMC11289207 39080184

[14] Løkkegaard T Todsen T Nayahangan LJ Point-of-care ultrasound for general practitioners: a systematic needs assessment Scand J Prim Health Care 2020 38 01 3 11 10.1080/02813432.2020.1711572 31955658 PMC7054965

[15] Schäfer WL Boerma WG Kringos DS QUALICOPC, a multi-country study evaluating quality, costs and equity in primary care BMC Fam Pract 2011 12 115 10.1186/1471-2296-12-115 22014310 PMC3206822

[16] Kringos D Boerma W Bourgueil Y The strength of primary care in Europe: an international comparative study Br J Gen Pract 2013 63 616 e742 50 10.3399/bjgp13X674422 24267857 PMC3809427

[17] Michels NRM Maagaard R Buchanan J Educational training requirements for general practice/family medicine specialty training: recommendations for trainees, trainers and training institutions Educ Prim Care 2018 29 06 322 326 10.1080/14739879.2018.1517391 30207897

[18] Mengel-Jørgensen T Jensen MB Variation in the use of point-of-care ultrasound in general practice in various European countries. Results of a survey among experts Eur J Gen Pract 2016 22 04 274 277 10.1080/13814788.2016.1211105 27487159

[19] Mulder TA van de Velde T Dokter E Unravelling the skillset of point-of-care ultrasound: a systematic review Ultrasound J 2023 15 01 19 10.1186/s13089-023-00319-4 37074526 PMC10115919

[20] Andersen CA Holden S Vela J Point-of-Care Ultrasound in General Practice: A Systematic Review Ann Fam Med 2019 17 01 61 69 10.1370/afm.2330 30670398 PMC6342599

[21] Myklestul HC Skjeie H Brekke M “Shades of grey”: a focus group study on diagnostic uncertainty among general practitioners using point-of-care ultrasound Scand J Prim Health Care 2024 1 11 10.1080/02813432.2024.2423242 PMC11834800 39503445

[22] Andersen CA Brodersen JB Mainz J Does point-of-care ultrasound examination by the general practitioner lead to inappropriate care? A follow-up study Scand J Prim Health Care 2025 1 13 10.1080/02813432.2025.2487095 PMC12377149 40207775

[23] Andersen CA Guetterman TC Fetters MD General Practitioners’ Perspectives on Appropriate Use of Ultrasonography in Primary Care in Denmark: A Multistage Mixed Methods Study Ann Fam Med 2022 20 03 211 219 10.1370/afm.2795 35606122 PMC9199035

[24] Osterwalder J Tabakovic S Jenssen C Emergency Point-of-Care Ultrasound Stewardship – A Joint Position Paper by EuSEM and EFSUMB and Endorsed by IFEM and WFUMB Ultraschall in Med 2023 44 04 379 388 10.1055/a-2077-7739 36996862

[25] Jarman RD Colclough A McDermott C EFSUMB Clinical Practice Guidelines for Point-of-Care Ultrasound: Part One (Common Heart and Pulmonary Applications) Ultraschall in Med 2023 44 01 36 49 10.1055/a-1882-6116 36228630

[26] Soni NJ Schnobrich D Mathews BK Point-of-Care Ultrasound for Hospitalists: A Position Statement of the Society of Hospital Medicine J Hosp Med 2019 14 E1 E6 10.12788/jhm.3079 30604779 PMC8021128

[27] Aakjær Andersen C Brodersen J Davidsen AS Use and impact of point-of-care ultrasonography in general practice: a prospective observational study BMJ Open 2020 10 09 e037664 10.1136/bmjopen-2020-037664 PMC7500300 32948563

[28] Lo H Frauendorf V Wischke S Ambulatory use of Handheld Point-of-Care Ultrasound (HH-POCUS) in rural Brandenburg – a pilot study Ultraschall in Med 2022 43 584 591 10.1055/a-1354-5958 33626573

[29] Aakjær Andersen C Jensen MBB Toftegaard BS Primary care physicians’ access to in-house ultrasound examinations across Europe: a questionnaire study BMJ Open 2019 9 09 e030958 10.1136/bmjopen-2019-030958. PMC6773286 31575576

[30] Akanuwe JNA Siriwardena AN Bidaut L Practitioners’ views on community implementation of point-of-care ultrasound (POCUS) in the UK: a qualitative interview study BMC Health Serv Res 2023 23 01 84 10.1186/s12913-023-09069-4 36698100 PMC9876652

[31] Andersen CA Brodersen JB Graumann O Factors affecting point-of-care ultrasound implementation in general practice: a survey in Danish primary care clinics BMJ Open 2023 13 10 e077702 10.1136/bmjopen-2023-077702 37848298 PMC10582891

[32] Ludden-Schlatter A Kruse RL Mahan R Point-of-Care Ultrasound Attitudes, Barriers, and Current Use Among Family Medicine Residents and Practicing Physicians PRiMER 2023 7 13 10.22454/PRiMER.2023.967474 PMC10351427 37465839

[33] Wüstner M Radzina M Calliada F Professional Standards in Medical Ultrasound – EFSUMB Position Paper (Short Version) – General Aspects Ultraschall Med 2022 43 05 456 463 10.1055/a-1854-2936 35850146

[34] EFSUMB 5 Year Strategic Plan https://efsumb.org/wp-content/uploads/2024/07/5-year-plan-July2024_update.pdf

[35] EFSUMB Minimum training recommendations for the practice of medical ultrasound Ultraschall in Med 2006 27 01 79 105 10.1055/s-2006-933605 16508866

[36] The British Medical Ultrasound Society and The Royal College of Radiologists. Recommendations for specialists practising ultrasound independently of radiology departments 2023 https://www.rcr.ac.uk/our-services/all-our-publications/clinical-radiology-publications/recommendations-for-specialists-practising-ultrasound-independently-of-radiology-departments-safety-governance-and-education/

[37] Andersen CA Løkkegaard T Nayahangan LJ A core curriculum of point-of-care ultrasound examinations for frontline physicians in primary care: results from a European Delphi study Ultraschall in Med 2025 10.1055/a-2590-5242 PMC13065360 40239950

[38] Andersen CA Hedegård HS Løkkegaard T Education of general practitioners in the use of point-of-care ultrasonography: a systematic review Fam Pract 2021 38 04 484 494 10.1093/fampra/cmaa140 33367766

[39] Andersen CA Jensen MB Structured POCUS education for primary care physicians: the transition from short workshops to sustainable longitudinal learning Educ Prim Care 2024 1 8 10.1080/14739879.2024.2395403 39607076

[40] Andersen CA Espersen M Brodersen J Learning strategies of general practitioners striving to achieve point-of-care ultrasound competence: a qualitative study Scand J Prim Health Care 2022 40 01 67 77 10.1080/02813432.2022.2036483 35166178 PMC9090417

[41] Kang SY Jo IJ Heo S Emergency medicine residents’ learning curve in diagnosing deep vein thrombosis with 3-point venous point-of-care ultrasound Int J Emerg Med 2024 17 75 10.1186/s12245-024-00645-x PMC11181670 38886639

[42] Tolsgaard MG Todsen T Sorensen JL International multispecialty consensus on how to evaluate ultrasound competence: a Delphi consensus survey PloS one 2013 8 02 e57687 10.1371/journal.pone.0057687 23469051 PMC3585207

[43] Hofer M Kamper L Miese F Quality indicators for the development and didactics of ultrasound courses in continuing medical education Ultraschall in Med 2012 33 01 68 75 10.1055/s-0031-1281649 21894600

[44] Noerholk LM Morcke AM Kulasegaram K Does group size matter during collaborative skills learning? A randomised study Med Educ 2022 56 06 680 689 10.1111/medu.14791 PMC9313549 35262226

[45] Landry A Eicken J Dwyer K Ten Strategies for Optimizing Ultrasound Instruction for Group Learning Cureus 2017 9 04 e1129 10.7759/cureus.1129 28473947 PMC5413361

[46] Pietersen PI Konge L Madsen KR Development of and Gathering Validity Evidence for a Theoretical Test in Thoracic Ultrasound Respiration 2019 98 03 221 229 10.1159/000500146 31137031

[47] Harden RM What is a spiral curriculum? Medical Teacher 1999 21 02 141 143 10.1080/01421599979752 21275727

[48] Damewood SC Leo M Bailitz J Tools for Measuring Clinical Ultrasound Competency: Recommendations From the Ultrasound Competency Work Group AEM Educ Train 2019 4 1 S106 S112 10.1002/aet2.10368 PMC7011402 32072114

